# *Pseudomonas aeruginosa* biofilm matrix polysaccharide Psl is regulated transcriptionally by RpoS and post-transcriptionally by RsmA

**DOI:** 10.1111/j.1365-2958.2010.07320.x

**Published:** 2010-09-02

**Authors:** Yasuhiko Irie, Melissa Starkey, Adrianne N Edwards, Daniel J Wozniak, Tony Romeo, Matthew R Parsek

**Affiliations:** 1 Department of Microbiology, University of WashingtonSeattle, WA 98195, USA; 2 Department of Microbiology, University of IowaIowa City, IA 52242, USA; 3 Department of Microbiology and Immunology, Emory UniversityAtlanta, GA 30322, USA; 4 Division of Infectious Disease, Center for Microbial Interface Biology, The Ohio State UniversityColumbus, OH 43210, USA; 5 Department of Microbiology & Cell Science, University of FloridaGainesville, FL 32611, USA

## Abstract

Extracellular polysaccharides are important components of biofilms. In non-mucoid *Pseudomonas aeruginosa* strains, the Pel and Psl polysaccharides are major structural components of the biofilm matrix. In this study, we demonstrate that the alternative σ-factor RpoS is a positive transcriptional regulator of *psl* gene expression. Furthermore, we show that *psl* mRNA has an extensive 5′ untranslated region, to which the post-transcriptional regulator RsmA binds and represses *psl* translation. Our observations suggest that upon binding RsmA, the region spanning the ribosome binding site of *psl* mRNA folds into a secondary stem-loop structure that blocks the Shine–Dalgarno sequence, preventing ribosome access and protein translation. This constitutes a novel mechanism for translational repression by this family of regulators.

## Introduction

*Pseudomonas aeruginosa* is found in a variety of environmental niches and can cause chronic, as well as acute infections in humans. Cystic fibrosis (CF) patients' airways are chronically infected with *P. aeruginosa* exhibiting the biofilm mode of growth.

Microbial biofilms are surface-associated microorganisms encased within an extracellular matrix. Extracellular polysaccharides are key components of the matrix, critical for building and maintaining biofilm structure (Sutherland, 2001; Branda *et al*., 2005). *P. aeruginosa* produces at least three different extracellular polysaccharides that can contribute to the matrix: alginate, Pel and Psl (Ryder *et al*., 2007). Mucoid variants that overproduce alginate are predominant colony morphotypes usually isolated from older CF patients (Govan and Deretic, 1996). Alginate is an important biofilm matrix component in mucoid strains (Hentzer *et al*., 2001). In non-mucoid strains, Pel and Psl polysaccharides have been shown to be critical for biofilm formation (Friedman and Kolter, 2004; Jackson *et al*., 2004; Matsukawa and Greenberg, 2004). Colony morphology variants that overproduce Pel and Psl have also been isolated from CF patients (Drenkard and Ausubel, 2002; Kirisits *et al*., 2005; Smith *et al*., 2006). These variants are called rugose small colony variants (RSCVs) and are characterized by a small, wrinkly appearance on solid medium. The prevalence of both mucoid and RSCV phenotypes in the CF environment highlights the importance of alginate, Pel and Psl for chronic infection and further suggests the importance of biofilm growth in this environment.

In contrast to alginate expression, whose regulation has been a focus of research for some time, we know very little regarding the regulation of *pel* and *psl* expression. Recent studies have described the transcriptional regulation of *pel* and *psl* by a secondary messenger molecule c-di-GMP and a transcription factor FleQ (Hickman and Harwood, 2008). Furthermore, c-di-GMP was recently reported to bind and allosterically regulate the activity of PelD, activating Pel synthesis and secretion (Lee *et al*., 2007). Finally, quorum sensing has been suggested to positively regulate *pel*and *psl* expression (Sakuragi and Kolter, 2007; Gilbert *et al*., 2009).

There are many examples of transcriptional control of extracellular polysaccharide genes in various species. There is also evidence for control at the level of protein translation. One example is the negative control of poly-β-1,6-*N*-acetyl-D-glucosamine (PGA) expression by the regulator CsrA in *Escherichia coli* (Wang *et al*., 2005). PGA is an extracellular polysaccharide important for biofilm formation in *E. coli*(Itoh *et al*., 2008). The CsrA/RsmA family of RNA binding proteins has been described in a variety of bacterial species to regulate protein translation (Lapouge *et al*., 2008; Babitzke *et al*., 2009). All known examples of CsrA/RsmA-mediated translational repression involve the binding of this protein to the mRNA leader and/or proximal coding region, thus blocking the access of ribosomes to the translation initiation region (Baker *et al*., 2002; Dubey *et al*., 2003; Wang *et al*., 2005, 2007; Lapouge *et al*., 2007). Previous studies of *rsmA* mutant strains of *P. aeruginosa* revealed possible involvement of RsmA in regulatory pathways important for quorum sensing, virulence, biofilm formation and motility (Pessi *et al*., 2001; Heurlier *et al*., 2004; Mulcahy *et al*., 2006, 2008). Whether these regulatory influences are due to direct regulation by RsmA or pleiotropic effects remains to be demonstrated.

In this study, we demonstrate that *psl* expression is controlled transcriptionally and translationally through two independent mechanisms. First we show that the stationary-phase σ-factor RpoS is a transcription factor that positively regulates *psl*expression. Strains lacking *rpoS* exhibit reduced *psl*transcripts and products. Overexpression of *rpoS* results in elevated *psl* transcription and an RSCV colony morphology on solid medium, which is a phenotype consistent with Psl overproduction. We also demonstrate that the *psl* transcript contains a large 5′ untranslated region (UTR). We then show that the post-transcriptional regulator RsmA directly binds to sequences within the 5′ UTR of the *psl* mRNA, repressing translation of *pslA*. We propose a novel mechanism for RsmA control that involves RsmA stabilization of a stem-loop structure in the mRNA that blocks ribosome access to the *pslA* Shine–Dalgarno (SD) sequence.

## Results

### Determining the transcriptional start site of the *psl* operon

**Fig. 1:**
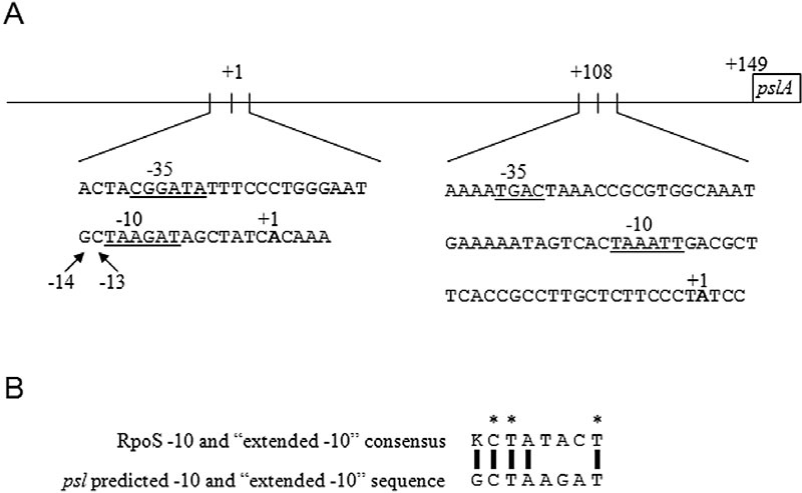
Two possible transcriptional start sites of *psl*.A. Transcriptional start site (+1 position) was determined by 5′ RACE analysis. Predicted RpoS/σ^S^-like −10 and −35 sites are underlined. The ‘extended −10 region’, a hallmark of σ^S^-dependent promoters, is denoted by positions −13 and −14 as indicated by arrows. A previously reported transcriptional start site is positioned 108 bp downstream of the +1 (Overhage *et al*., 2005). σ^70^-like −10 and −35 sites predicted by Overhage *et al*. are also underlined. *pslA* ORF is boxed, and the first nucleotide position of *pslA* is labelled as position +149.B. Sequence alignment of RpoS-dependent −10 and ‘extended –10’ consensus compiled from previous reports (Schuster *et al*., 2004; Typas *et al*., 2007) and the predicted *psl*−10 and ‘extended –10’ sequence (K = T/G). Nucleotides that were 100% conserved in all examined RpoS-dependent promoters by Schuster *et al*. are labelled with asterisks.

To initiate our study of the regulation of *psl*expression, we first performed 5′ RACE. The transcriptional start site was found to be 148 bp upstream of the *pslA* open reading frame (ORF) (Fig. 1A). Interestingly, this result was not consistent with a previous study that also employed 5′ RACE (Overhage *et al*., 2005). Overhage *et al*. reported the transcriptional start site to be 41 bp upstream of the ATG of *pslA* (Fig. 1A). They predicted the corresponding −10 and −35 promoter regions resembled the consensus sequence for σ^70^. However, the predicted promoter sequences are positioned with the −10 region centred approximately 30 bp upstream of their transcriptional start site. This was unusual as the typical distance between the +1 and −10 of a σ^70^-dependent promoter is 5–8 bp (Typas *et al*., 2007). Culturing conditions between the two studies were identical [PAO1 grown in Luria–Bertani (LB) to an OD_600_ of 1.0], although the use of different RACE primers could potentially contribute to the differences observed in the two studies.

**Fig. 2:**
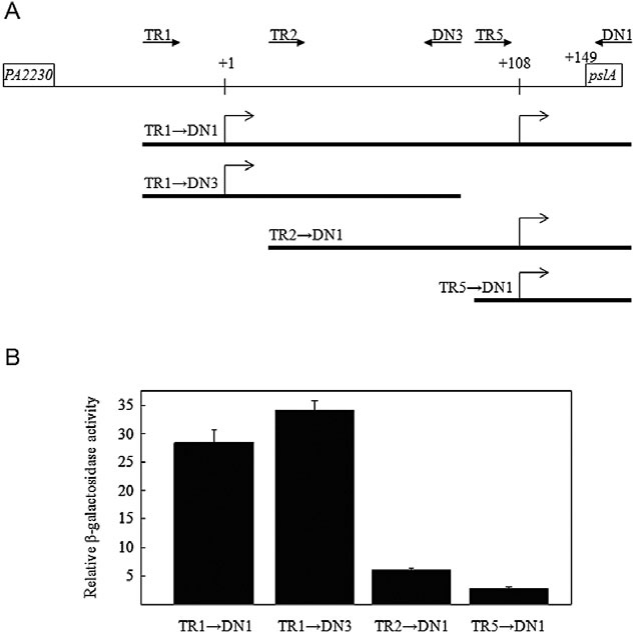
Transcriptional fusion studies confirm the 5′ RACE-derived transcriptional start site of *psl*.A. Transcriptional *lacZ* fusion constructs of *psl* promoter region. Four representative transcriptional fusion constructs span the corresponding regions as indicated by the black bars, with the locations of the two putative transcriptional start sites. The approximate positions of the TR1, TR2, TR5, DN1 and DN3 primers that were used to construct the transcriptional fusions are shown as horizontal arrows.B. Relative expression of the four representative transcriptional fusion constructs. The results indicate that the promoter associated with the +1 transcriptional start site has the highest activity instead of the previously published +108 transcriptional start position. The *y*-axis unit is described as β-galactosidase activity determined by the Galacto-Light Plus kit divided by total mg of protein from the cell lysates as determined by Bradford assay.

To investigate the discrepancies in the results, we designed a series of nested transcriptional fusion reporter constructs that spanned various regions upstream of *pslA*. The representative constructs are displayed in Fig. 2A. As shown in Fig. 2B, the reporters containing the promoter region from our study (TR1→DN1 and TR1→DN3) had significantly higher transcriptional activity compared with the reporter containing only the promoter reported in the Overhage study (TR2→DN1 and TR5→DN1). While it is possible that *psl* has two distinct promoters, we conclude that the majority of the *psl* transcripts initiate at 148 bp upstream of *pslA* ORF (designated as +1 in this paper).

### RpoS transcriptionally regulates *psl* expression

**Fig. 3:**
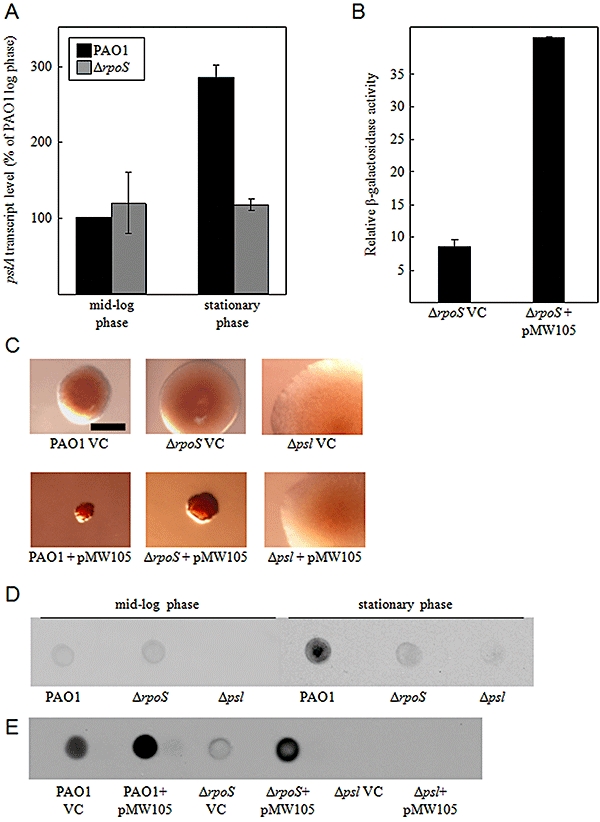
*psl* transcripts are regulated by RpoS.A. *pslA* transcript levels were measured using quantitative real-time PCR. Transcripts increased in stationary phase compared with mid-log phase in PAO1, but did not increase in Δ*rpoS*.B. Mid-log phase grown cells constitutively overexpressing RpoS by the introduction of pMW105 increased *psl* transcription compared with the pEX1.8 vector control (VC) strains. The *y*-axis unit is described as β-galactosidase activity divided by total mg of protein from the cell lysates.C. Strains constitutively overexpressing RpoS from pMW105 confer RSCV phenotypes compared with smooth colonies of pEX1.8 VC strains when grown on VBMM Congo Red plates. Scale bar = 0.5 mm.D. α-Psl immunoblot indicated an increase in Psl production by PAO1 in stationary phase, but not by Δ*rpoS*.E. α-Psl immunoblot demonstrated an increase of Psl production by RpoS overexpression strains compared with their respective vector control strains in PAO1 and Δ*rpoS*.

In *P. aeruginosa*, RpoS is an alternative σ-factor that regulates expression of a number of genes in the stationary phase (Schuster *et al*., 2004). Schuster *et al*. observed that all the genes in the *psl* operon (*pslA*-*L*) were upregulated during stationary-phase growth in wild-type (WT) PAO1, but not in an isogenic Δ*rpoS* mutant strain. We therefore tested whether RpoS controls *psl* transcription. As shown by quantitative real-time polymerase chain reaction (PCR) in Fig. 3A, *psl* transcripts increased about threefold in stationary phase compared with mid-log phase in WT cultures, but Δ*rpoS* failed to show an increase in *psl* transcription during stationary phase. To confirm a functional consequence for the RpoS-dependent increase in *psl* transcription in stationary phase, we analysed relative Psl levels using Psl-specific antisera (Fig. 3D). As predicted from the quantitative real-time PCR data, the amount of Psl produced by WT and Δ*rpoS* were low during mid-log phase, and only WT increased during stationary phase.

To further investigate the involvement of RpoS in *psl* expression, we overexpressed RpoS and observed that *psl* transcription increased significantly (Fig. 3B). Consistent with previous work examining the effects of *psl*overexpression (Ma *et al*., 2006), strains overexpressing RpoS exhibited the RSCV morphology (Fig. 3C) and increased Psl production (Fig. 3E). The RSCV phenotype is lost when RpoS is overexpressed in a Δ*psl*background, verifying that the RSCV phenotype is due to Psl production.

We also observed that our newly predicted promoter sequences of the *psl* operon strongly resemble that of an RpoS/σ^S^-dependent promoter. The −10 region of *psl* aligned well with the previously derived consensus sequence (identical to that of *E. coli*) for the *P. aeruginosa* RpoS-dependent promoters (Fig. 1B) (Schuster *et al*., 2004). The ‘extended −10 region’, known as the −13 and −14 positions, is a highly conserved hallmark of σ^S^-dependent promoters in *E. coli* that distinguishes them from σ^70^ promoters (Typas *et al*., 2007). The *E. coli* consensus for the −13 position is a C, and a G/T for the −14 position (Barne *et al*., 1997). Our predicted *psl*−10 sequence is a perfect match to the consensus motif (Fig. 1B).

### *ΔrsmA* mutations confer a Psl-dependent RSCV phenotype

**Fig. 4:**
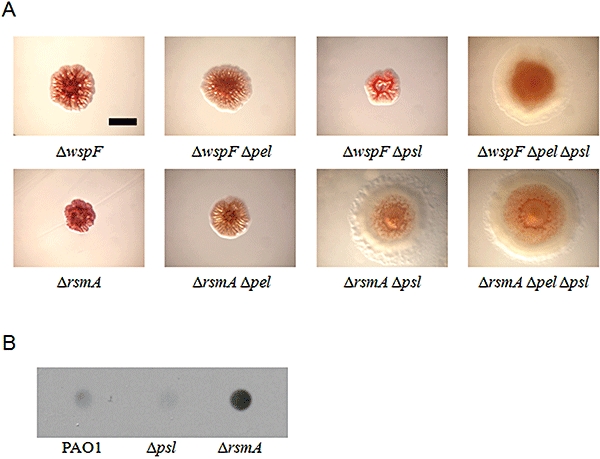
Δ*rsmA* RSCV phenotype is Psl-dependent.A. The RSCV phenotype is only relieved when both *pel* and *psl* loci are mutated in the Δ*wspF* background, while a *psl* mutation is sufficient to confer smooth colony morphology in the Δ*rsmA* background. All strains were streaked on VBMM Congo Red plates. Scale bar = 1 mm.B. Psl polysaccharides extracted from overnight cultures of PAO1 WT, Δ*psl* and Δ*rsmA* were detected by performing α-Psl immunoblot. Δ*rsmA* extracts displayed significantly greater Psl production than PAO1.

Previous work on the RSCV phenotype has linked it to elevated *pel* and *psl* expression (Friedman and Kolter, 2004; Hickman *et al*., 2005; Kirisits *et al*., 2005; Ueda and Wood, 2009; Malone *et al*., 2010). In the well-characterized Δ*wspF* RSCV background, this mutation results in the hyper-activation of WspR, producing elevated levels of c-di-GMP (Hickman *et al*., 2005). This results in enhanced transcription of the *pel* and *psl* operons (Hickman *et al*., 2005; Kirisits *et al*., 2005). Mutations in both *pel* and *psl* in a Δ*wspF* strain convert the RSCV phenotype to a WT smooth colony (Fig. 4A) while no colony morphology changes are observed when *pel* and *psl* are mutated in the WT background (Fig. S1).

We observed that Δ*rsmA* mutant strains produced a RSCV colony morphology similar to that of Δ*wspF* (Fig. 4A). We therefore hypothesized that both Pel and Psl contribute to the RSCV phenotype in the Δ*rsmA* strain. However, unlike Δ*wspF*, a Δ*rsmA* mutant strain only required a *psl* mutation to convert the RSCV to a smooth phenotype (Fig. 4A). This observation suggested that only Psl contributes to the RSCV phenotype of a Δ*rsmA* strain and that Psl expression is elevated in this background. We subsequently confirmed elevated Psl levels in a Δ*rsmA* strain by performing α-Psl immunoblots (Fig. 4B).

**Fig. 5:**
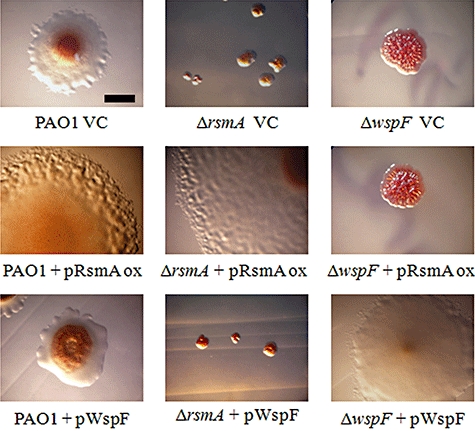
RsmA and WspF mutations represent independent pathways producing RSCV phenotypes. RsmA overexpression (pRsmA ox) does not affect the RSCV phenotype of Δ*wspF*, nor does WspF overexpression (pWspF) in Δ*rsmA*. Scale bar = 1 mm. VC, vector control (pUCP18).

Differences in the Pel and Psl dependence of the RSCV phenotypes in Δ*rsmA* and Δ*wspF* strains suggested that the RSCV formation followed separate molecular mechanisms. RsmA overexpression in a Δ*wspF* background and WspF overexpression in a Δ*rsmA* background failed to reverse the RSCV phenotypes to WT colony morphologies (Fig. 5), further supporting that Wsp-dependent and RsmA-dependent RSCV pathways are epistatically independent of each other.

### *psl* translation, not transcription, is elevated in a *ΔrsmA* mutant strain

As Psl expression was elevated in a Δ*rsmA*strain, we hypothesized that RsmA was acting as a translational repressor of *psl*expression. In order to test this, we generated *pslA* chromosomal transcriptional and translational *lacZ* fusion reporter strains. We compared expression of *psl*transcription and translation in stationary-phase cultures of a WT and Δ*rsmA*.

**Fig. 6:**
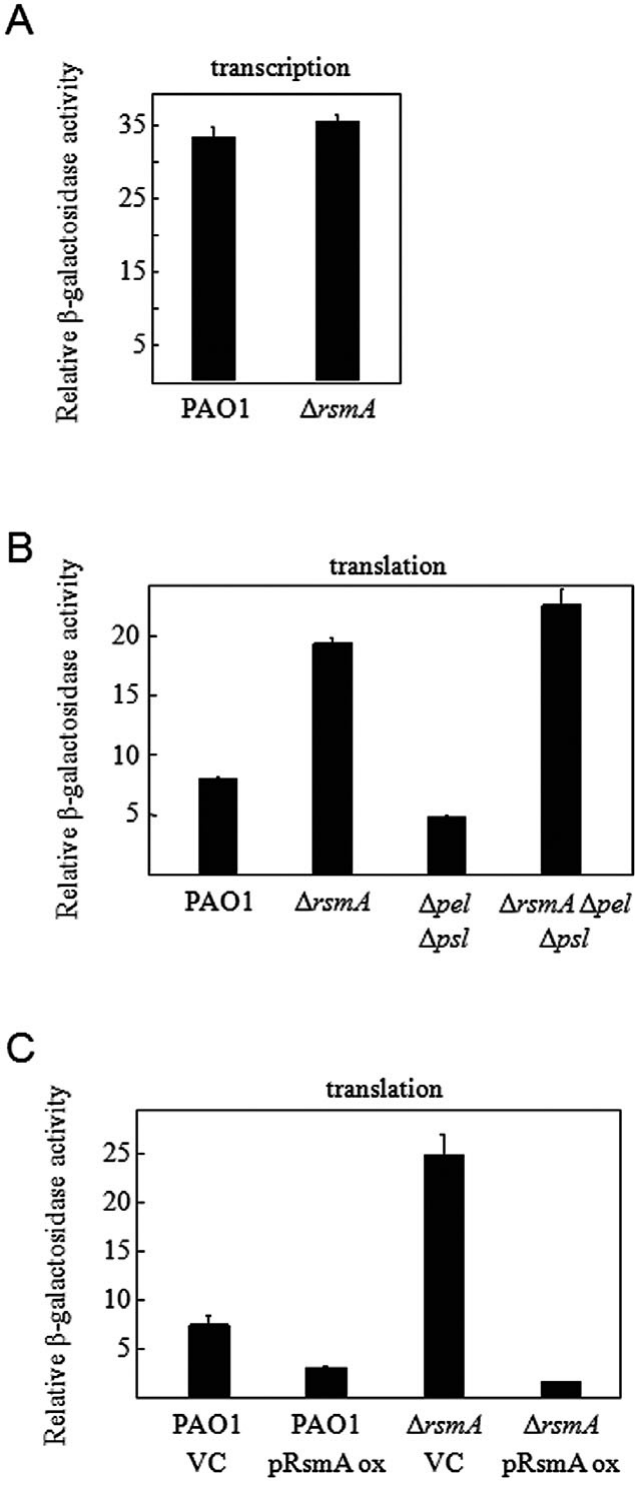
RsmA is a post-transcriptional regulator of *psl*.A. Transcriptional *lacZ* fusion constructs in WT and Δ*rsmA* backgrounds reveal no significant differences in *psl* transcription.B. Translational *lacZ* fusion constructs in WT and Δ*rsmA* backgrounds show an increase of *psl* translational activity in the Δ*rsmA* background. RsmA regulation of *psl* is independent of the expression of Pel and Psl as indicated by the increase of *psl* translation in Δ*rsmA*Δ*pel*Δ*psl* compared with Δ*pel*Δ*psl*, suggesting that this is not an effect from the autoaggregation seen in the delta rsmA strain.C. Translational *lacZ* fusion constructs in WT and Δ*rsmA* backgrounds with pUCP18 vector control (VC) and RsmA overexpression plasmid (RsmA ox) result in a decrease of *psl* translation when RsmA is overexpressed.

Translational fusion data showed that *psl* translation was threefold to fourfold higher in Δ*rsmA* than WT (Figs 6B and S2B), despite *psl* transcriptional activity being similar (Figs 6A and S2A). Elevated *psl* translational activity in Δ*rsmA* could be complemented by overexpressing RsmA (Fig. 6C). RsmA overexpression further reduced the activity below the WT level, providing additional evidence that RsmA acts as a repressor of *psl*.

**Fig. 7:**
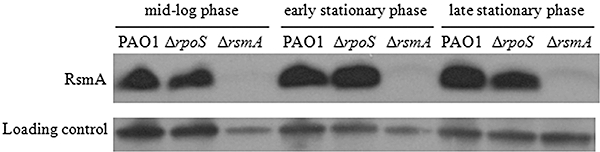
RsmA is not regulated by RpoS. Western blot of PAO1, Δ*rpoS* and Δ*rsmA* cell lysates was performed using α-*E. coli* CsrA antibodies. Cells were grown in LB and harvested at mid-log phase (*t* = 3.5 h. post-inoculation, OD_600_ = 1.0), early stationary phase (*t* = 6.5 h., OD_600_ = 4.0) and late stationary phase (*t* = 9 h., OD_600_ = 4.0). Total protein concentration was normalized before loading onto an 18% Tris-HCl SDS polyacrylamide gel. PAO1 and Δ*rpoS* did not demonstrate significant differences in RsmA protein levels by Western blot analysis. Loading control shown is a non-specific reactive band to the α-CsrA antibodies present in all cell lysates.

Previous reports have concluded that RsmA expression is elevated in stationary phase (Pessi *et al*., 2001; Brencic and Lory, 2009). This is somewhat paradoxical, as RpoS activates *psl* transcription maximally at stationary phase, while RsmA represses *psl* translation. Therefore we sought to discern whether RsmA levels are influenced by RpoS. Western blot analyses revealed no substantial changes of RsmA expression levels in Δ*rpoS* compared with WT (Figs 7 and S3). Furthermore, the levels of RsmA protein did not vary significantly over the course of growth of either strains, inconsistent with the aforementioned previous studies. We conclude that RpoS does not regulate RsmA, and that *psl* transcriptional and translational regulation by RpoS and RsmA respectively, represent two distinct modes of control.

### Characterization of RsmA binding site to the 5′ UTR of *psl* mRNA

**Fig. 8:**
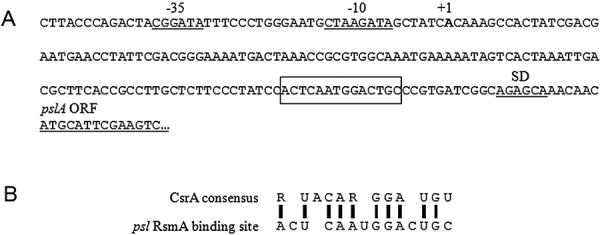
Predicted RsmA binding site in the *psl* 5′ UTR.A. The predicted position of SD is indicated by the underlined 7–12 bases upstream of the start codon of *pslA* ORF. Predicted RsmA binding site is boxed, 24–37 bases upstream of the start codon. The asterisks indicate the GG nucleotide pair required for CsrA/RsmA binding to its RNA target, which was mutated to the CC pair for the experiments demonstrated in Fig. 11. +1 indicates the transcriptional start site (in bold font) and the corresponding −10 and −35 regions of the promoter (underlined).B. Sequence alignment of *psl* RsmA binding site with the CsrA consensus sequence (R = A or G) as previously reported (Dubey *et al*., 2005).

*P. aeruginosa* RsmA and *E. coli* CsrA share 85.2% sequence identity, and heterologous expression of *P. aeruginosa* RsmA has previously been shown to complement an *E. coli csrA* mutant (Pessi *et al*., 2001). Thus, it appears likely that RsmA binding sites in *P. aeruginosa* mRNAs should resemble the *E. coli* CsrA consensus sequence generated by SELEX and other studies (Baker *et al*., 2002; Dubey *et al*., 2003, 2005; Gutiérrez *et al*., 2005; Wang *et al*., 2005). We identified only one probable RsmA binding site within the 5′ UTR of the *psl* transcript, spanning the region 24–37 bases upstream of the start codon (Fig. 8) and 12–25 bases upstream of the predicted SD sequence.

**Fig. 11:**
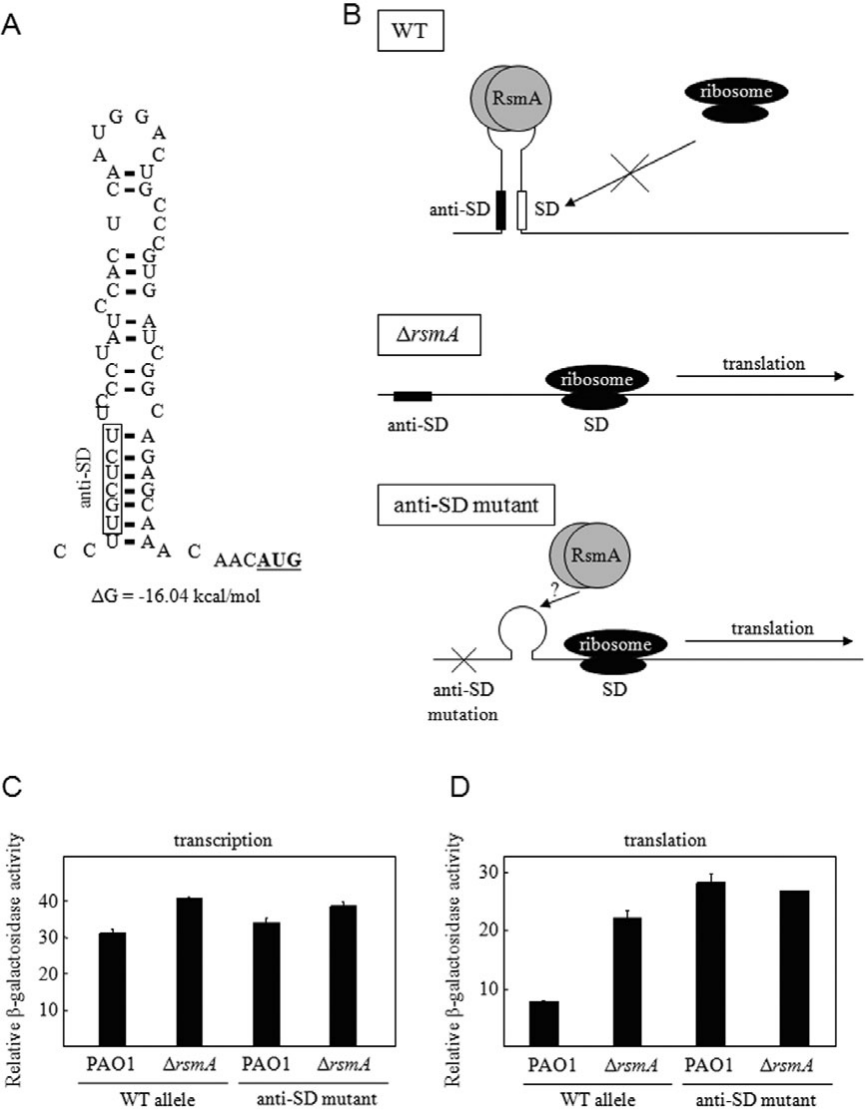
RsmA binding to the 5′ UTR of *psl* mRNA may promote double-stranded RNA binding between SD and anti-SD sequences.A. Possible two-dimensional structure of *psl* mRNA translational control region predicted by mFOLD. The translational start codon is shown in bold font and underlined. The RsmA binding site is located in the top portion of the stem-loop structure, with the essential GG pair in the loop region. The anti-SD sequence (UGCUCU) is boxed. The anti-SD sequence was mutated to CUGCAG (anti-SD mutant) in order to prevent its base-pairing with the SD sequence.B. Representative images of RsmA–*psl* repression model. In WT cells, RsmA homodimers bind to the RsmA binding site located in the 5′ UTR of *psl* mRNA. This results in the formation of a stem-loop structure, which base-pairs the SD sequence with the anti-SD sequence. The double-stranded RNA form blocks ribosome access to the SD site, preventing ribosome assembly and therefore inhibiting translation of *psl*. In the Δ*rsmA* mutant, the stem-loop structure is not stable enough to prevent ribosome access to the SD site, therefore initiating *psl* translation. Constructs designed to prevent SD base-pairing (anti-SD mutant) may still allow RsmA binding to the 5′ UTR of *psl* resulting in a smaller stem-loop structure, but the SD site remains accessible for ribosome to initiate translation of *psl*.C. Transcriptional *lacZ* fusion constructs of the anti-SD mutant does not display altered levels of *psl* transcription in PAO1 and isogenic Δ*rsmA* backgrounds.D. Translational *lacZ* fusion constructs of the anti-SD mutant demonstrate an increase of *psl* translation compared with WT in PAO1 background, similar to the *psl* translation level of Δ*rsmA*. The stem disruption mutation did not significantly affect the levels of *psl* translation in the Δ*rsmA* background.

**Fig. 9:**
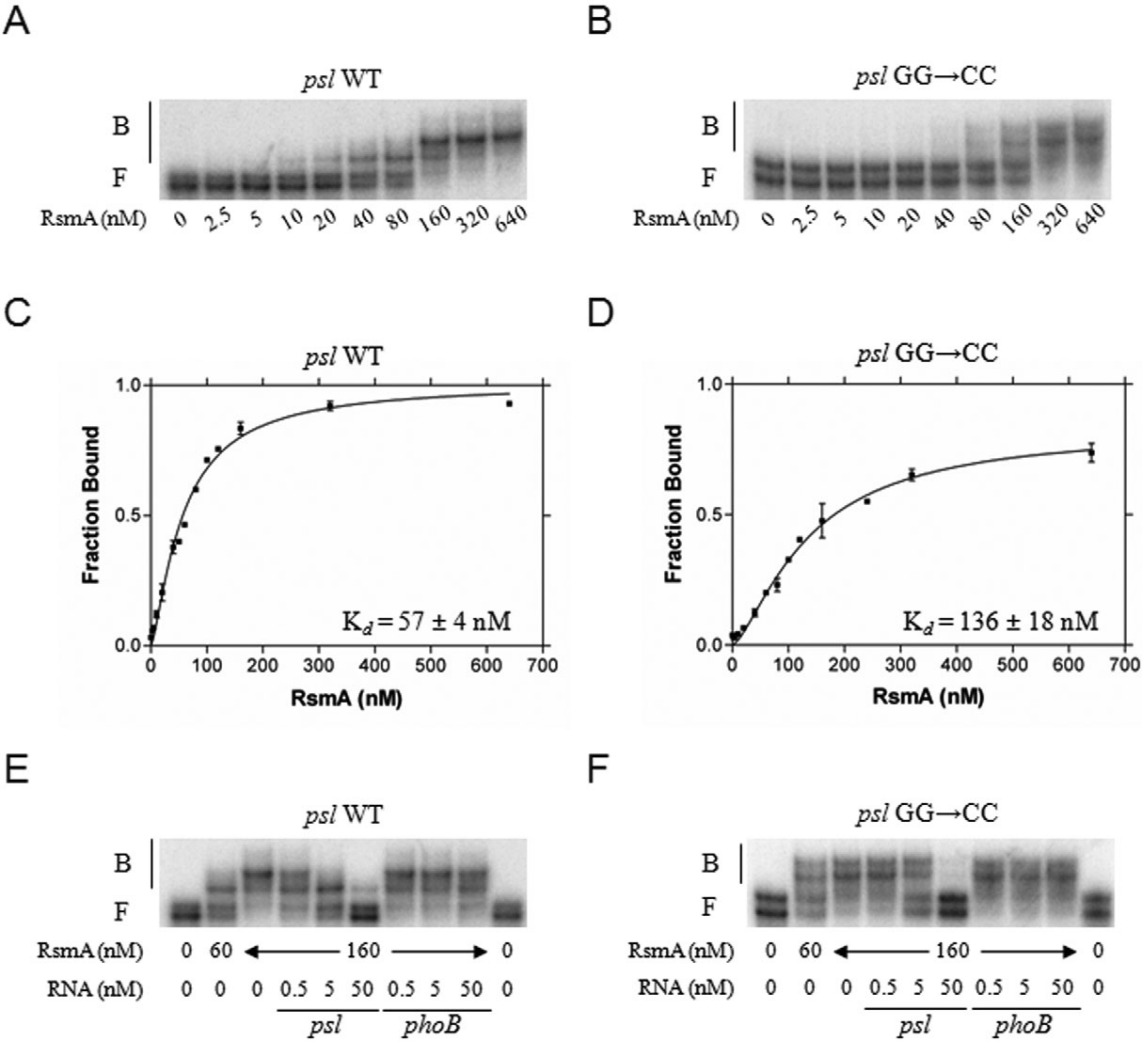
RsmA protein binds to the 5′ UTR of *psl* mRNA.Gel mobility shift analyses of RsmA–*pslA* (A) and RsmA–*pslA*GG→CC (B) interactions in the absence of RNA competitor. 5′-end labelled WT *pslA*or *pslA* GG→CC transcripts (50 pM) were incubated with RsmA at the indicated concentrations. The positions of free (f) and bound (b) RNA are shown. WT *pslA* RNA displays two shifts, while the mutated *pslA* GG→CC only has one apparent shift. The K*_d_* values of RsmA binding to the WT *pslA* RNA (C) and to the *pslA* GG→CC RNA (D) were calculated using non-linear least-square analysis of data from three independent gel mobility shift assays. Competition reactions using specific (*pslA* or *pslA* GG→CC) or non-specific (*phoB* from *E. coli*) unlabelled RNA competitors (E and F). The concentrations of RsmA and competitor RNA are shown at the bottom of the corresponding lanes.

To determine whether RsmA binds to the predicted binding site, we purified RsmA with a His_6_ C-terminal tag (RsmA–His_6_; Fig. S4) to perform RNA gel mobility shift assays. RsmA–His_6_ was demonstrated before the assay to be functional *in vivo*, as it could successfully complement Δ*rsmA* (Fig. S5). As seen in Fig. 9A, C and E, RsmA–His_6_ demonstrated specific, high affinity binding to the *psl* mRNA. Interestingly, two distinct complexes were formed. The slower moving complex appeared at RsmA concentrations higher than 160 nM (Fig. 9A). This raises the possibility that there is a second binding site on the *psl* mRNA, at which RsmA could bind at a lower affinity.

**Fig. 10:**
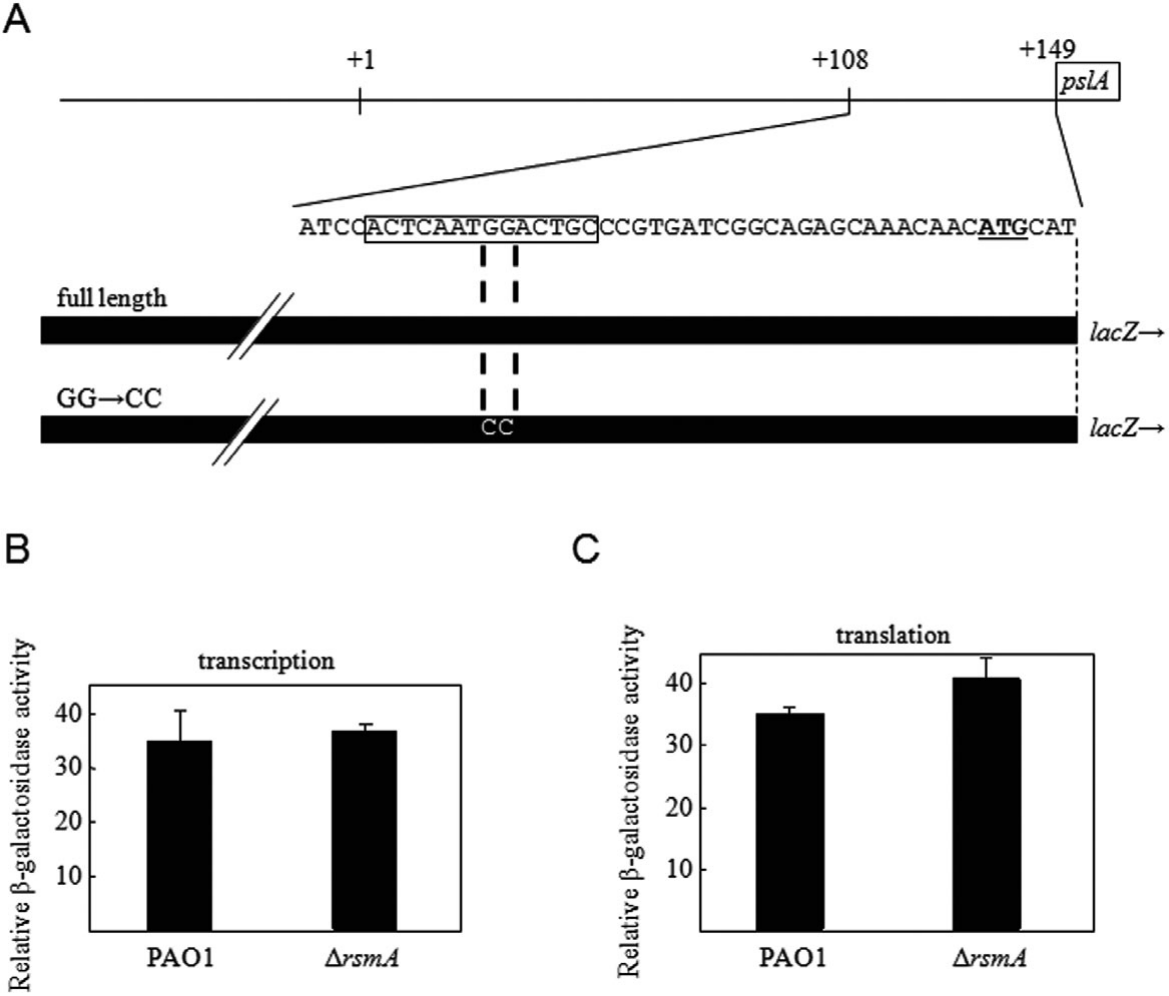
GG→CC mutation within the RsmA binding site abolishes RsmA-mediated regulation of *pslA* translation.A. Translational fusion constructs of full length and GG→CC constructs. The GG nucleotides in the RsmA binding site (boxed) important for RsmA binding were mutated to CC.B. Transcriptional and C. translational *lacZ* fusion constructs assayed for β-galactosidase activities show deregulation of *pslA* translation in the Δ*rsmA* compared with WT when GG pair was mutated into CC.

The GGA triplet that is centrally located in the consensus sequence is essential for high affinity CsrA binding (Dubey *et al*., 2005). We therefore constructed an altered *psl* mRNA where GG was replaced with CC (Fig. 10A). The GG→CC substitution abolished the fast moving RsmA–RNA complex (Fig. 9B, D and F), indicating that the predicted binding site was indeed a direct target of RsmA. The GG→CC mutation did not eliminate the secondary shift complex seen at 320 nM RsmA, suggesting that the putative second binding site is still functional in the absence of the high affinity binding site. Replacing the WT sequence with the GG→CC mutation in the translational and transcriptional reporter strains resulted in a loss of RsmA control of translation (Fig. 10C).

### Model for RsmA repression of *psl* translation

The CsrA/RsmA family typically represses gene expression by binding to the mRNA leader and directly blocking access of the ribosome to the ribosome binding site (RBS) (Lapouge *et al*., 2008). In addition, CsrA/RsmA-bound RNAs assume stem-loop secondary structures (Schubert *et al*., 2007). The high affinity RsmA binding site of *psl*, however, does not appear to overlap with the RBS, as is usually the case. We noted that there was an alternative potential translational start site GTG, located 21 bases upstream of the PAO1 genome annotated translational start site ATG, which would add 7 amino acids to the N-terminus of PslA without frameshifting the ORF (Fig. S6A). Furthermore, placing GTG as the translational start site would position the SD sequence overlapping with the experimentally determined RsmA binding site, similar to other known systems. In order to determine the true translational start site of *pslA*, we constructed fusion strains that were fused to *lacZ* immediately upstream of the AUG codon. The construct without AUG showed equal transcriptional activity (Fig. S6B) as the transcriptional fusion constructs contain a synthetic translational start site, but the translational fusion activity was abolished (Fig. S6C). These results demonstrate that the *psl* AUG codon is an indispensable element in translation, and suggest that the translational start site of *pslA* is the AUG codon, and not the GUG codon.

We noticed that there was a perfect inverted complement region to the SD sequence (termed ‘anti-SD sequence’ here) between 10 and 15 bases upstream of the RsmA binding site. Furthermore, prediction of the secondary structure of the *pslA* leader by mFOLD software (Zuker, 1989) revealed a large stem-loop structure in which the anti-SD sequence and the SD sequence are base-paired at the base of the stem (Fig. 11A). We hypothesized that RsmA binding to the target site on the *psl* mRNA stabilizes the large stem-loop structure, which sterically hinders the ribosome access to the SD sequence by base-pairing with the anti-SD sequence (Fig. 11B). We therefore attempted to destabilize this structure by mutating the anti-SD sequence UGCUCU into GACGUC. The mutation was predicted to prevent base-pairing and free the SD sequence to allow ribosome access, resulting in derepression of *psl* in the presence of RsmA (Fig. 11B). The anti-SD mutation completely alleviated RsmA repression of *psl* in the WT background, elevating *psl* translational activity to an identical level as that of Δ*rsmA* (Fig. 11D). Furthermore, *psl* translational activity of the anti-SD mutant was indistinguishable between WT and Δ*rsmA*, suggesting that RsmA repression of *psl* translation acts through the anti-SD sequence. This model of translational repression is, to our knowledge, novel for CsrA/RsmA regulatory mechanisms.

## Discussion

Extracellular polysaccharides comprise major components of the biofilm matrix in many bacterial species (Sutherland, 2001; Branda *et al*., 2005). In *P. aeruginosa* PAO1, Psl is an important component of the matrix. Without the capacity to produce Psl, PAO1 exhibits a profound attachment and biofilm development defect (Ma *et al*., 2006). Psl appears to be indispensable for biofilm formation, and understanding its regulation informs us about the environmental signals influencing biofilm formation. In this paper, we present evidence that Psl regulation is complex. We show that the *psl* operon is transcriptionally regulated by stationary-phase σ-factor RpoS and translationally by the post-transcriptional regulator RsmA. Furthermore, we demonstrate that these mechanisms of control are mediated independently of one another.

RpoS/σ^S^ regulation has previously been linked to biofilm formation in *P. aeruginosa* and other bacterial species (Adams and McLean, 1999; Xu *et al*., 2001; Corona-Izquierdo and Membrillo-Hernández, 2002). The prevalence of a slow-growing subpopulation within biofilm communities has been demonstrated numerous times for *P. aeruginosa* (Walters *et al*., 2003). An earlier study demonstrated that Psl was important not only for cell–surface attachment and cell–cell adhesion, but had to be actively produced in order to maintain biofilm structure (Ma *et al*., 2006). This might suggest that stationary-phase populations within the biofilm are important for producing Psl. This is supported by Overhage *et al*., who demonstrated that biofilm cells are transcriptionally expressing the *psl* operon (Overhage *et al*., 2005). Another key point is that transition to the stationary phase of growth by liquid cultures of *P. aeruginosa* may promote biofilm production through Psl expression. This indirectly suggests that a response to cessation of growth may be to coordinate initiation of biofilm formation, perhaps as a protection mechanism for starved cells. Intriguingly, a recent report placed a quorum sensing regulator LasR binding site upstream of the −35 region of *psl* (Gilbert *et al*., 2009). More detailed studies will need to be conducted in order to investigate whether LasR-mediated regulation involves RpoS.

We also provide evidence that *psl* is regulated post-transcriptionally by the RNA binding protein RsmA. This family of proteins has been shown to repress translation of target mRNAs by competing with the ribosome for binding to the mRNA leader (Babitzke *et al*., 2009). This often, though not invariably, results in decreased stability and steady state levels of target mRNAs. An exception to this trend is the *E. coli hfq* gene, which is translationally repressed without concomitant effects on its stability (Baker *et al*., 2007). We observed that RsmA post-transcriptionally represses *psl* without affecting transcription. We also noted that minor *psl* transcriptional changes (approximately twofold) due to a mutation in *rsmA* were documented in one recent microarray study (Brencic and Lory, 2009), but not in another (Burrowes *et al*., 2006). It is unclear why these inconsistencies exist.

In *E. coli* and many other species, small regulatory RNAs (sRNAs) CsrB and CsrC bind multiple copies of CsrA, and thereby sequester and antagonize its activity (Liu *et al*., 1997; Weilbacher *et al*., 2003). These non-coding RNAs require a two-component signal transduction system, BarA–UvrY, for transcription (Suzuki *et al*., 2002; Weilbacher *et al*., 2003), and are targeted for degradation by RNase E via CsrD (Suzuki *et al*., 2006). The BarA sensor kinase responds to metabolic by-products of glycolysis, including formate and acetate (Chavez *et al*., 2010). CsrA, in turn, activates glycolysis and downregulates gluconeogenesis and glycogen biosynthesis (Sabnis *et al*., 1995). Thus, CsrA both governs and responds to the metabolic status of the cell. The complex genetic feedback circuitry of this system permits CsrA to regulate expression of the *csrB*, *csrC* and *csrD* genes (Suzuki *et al*., 2006; Romeo and Babitzke, 2010). Elements of this circuitry are present and appear to function similarly in *Pseudomonas*species: a homologous two-component system GacS–GacA transcribes the sRNAs that antagonize RsmA (Valverde *et al*., 2004; Humair *et al*., 2010). Nevertheless, it does not appear that *Pseudomonas* species encode a conserved homologue for CsrD, and it is premature to conclude that the overall strategy for circuitry design is conserved in these species.

RsmA repression of *psl* mRNA is novel, in that the target binding site of RsmA does not appear to overlap the RBS. *E. coli* CsrA binds to six sites on the *pga* mRNA, two of which overlap the SD sequence and start codon, effectively blocking ribosome access (Wang *et al*., 2005). The high affinity RsmA binding site of *psl*, however, is located 12 bases upstream of the SD sequence. High affinity binding sites for CsrA are found in the single-stranded loop of a stem-loop structure (Dubey *et al*., 2005), and a recent structural study of RsmA homologue RsmE in complex with RNA revealed that protein binding induces the formation of a secondary stem-loop structure in the RNA (Schubert *et al*., 2007). mFold software analysis of the 5′ UTR region of *psl* predicted a large, imperfect stem-loop structure in which the RsmA binding site is located within the loop region, consistent with the binding properties of this protein. Furthermore, the predicted structure contains a double-stranded RNA base-pairing event between the SD sequence and an anti-SD sequence, which form the base of the stem (Fig. 11A). Disruption of the anti-SD–SD pairing led to an increase of *psl* translational activity, which was no longer responsive to RsmA (Fig. 11D). These findings suggest that the RsmA-mediated repression of *psl*translation involves the anti-SD sequence. One possible mechanism that can be envisioned is that RsmA binding helps to stabilize base-pairing between the SD and the anti-SD in the imperfect stem-loop. *PA0081*, *PA0082* and *PA4492* have recently been identified as direct targets of RsmA (Brencic and Lory, 2009). Unlike *pslA*, RsmA binding sites appear to overlap with either the start codon, putative SD sequence, or both, consistent with previous models in the literature for other described RsmA/CsrA targets. Without specific experimentation, we do not know the exact lengths of their 5′ UTRs and therefore cannot accurately rule out the possibility of similar anti-SD sequences, although there are no obvious anti-SD sequences upstream of the SD of these genes. It will be interesting to pursue whether anti-SD sequences are more common in RsmA/CsrA regulated genes in *P. aeruginosa* and other organisms.

Gel shift assays revealed that the presence of a possible second RsmA binding site within the leader transcript of *psl* (Fig. 9). Other than the primary binding site determined in this study (Figs 9 and 10), another GGA triplet is located 70 bases upstream of the RsmA binding site. While the flanking region does not share any homology with the SELEX-derived *E. coli* consensus sequence, we cannot rule out potential weak binding by RsmA to this region. In support of this possibility, it was recently demonstrated that dual-site binding of CsrA to target RNAs strengthens regulation. Molecular bridging of the CsrA homodimer occurs when a high affinity binding site is positioned adjacent to a lower affinity binding site on the mRNA (Mercante *et al*., 2009). Future experiments will explore the nature of this second binding site.

The ability to enter or leave the biofilm lifestyle would be vital to *P. aeruginosa*'s adaptations to new or changing environments. One way to regulate this lifestyle change would be through controlling the production of extracellular matrix components such as Psl. Our study indicates that there are at least two levels of Psl regulation. Clearly, transcriptional regulation by RpoS ties Psl expression to the growth phase of the cell. This suggests that cessation of active growth would promote biofilm formation. What is not so clear are the signals governing translation control of Psl by RsmA. As RsmA levels were not observed to differ dramatically over the growth curve, translational control of Psl expression may be subject to environmental signals controlling RsmY and RsmZ sRNA expression. Additionally, perhaps an increase in stationary-phase levels of *psl* mRNA exceeds the binding capacity of RsmA leading to a relief in translational repression. Unfortunately, the environmental signals controlling the Gac–RsmA system in *P. aeruginosa* are yet to be identified. Nevertheless, a more thorough understanding of how bacteria regulate biofilm matrix production may be the key for developing strategies targeting biofilms in industry and medicine.

## Experimental procedures

### Bacterial strains and growth conditions

The bacterial strains used in this study are listed in Table S1. *E. coli* and *P. aeruginosa* strains are propagated in LB medium at 37°C unless otherwise specified. Selective medium for *P. aeruginosa* used throughout this study is VBMM with citrate as the carbon source (Hoang and Schweizer, 1997). Concentrations of antibiotics used for *E. coli* were: 100 µg ml^−1^ ampicillin or 50 µg ml^−1^ carbenicillin, 10 µg ml^−1^ gentamicin and 10 µg ml^−1^ tetracycline. For *P. aeruginosa*: 300 µg ml^−1^ carbenicillin, 100 µg ml^−1^ gentamicin and 100 µg ml^−1^ tetracycline were used. Sucrose counter-selection for *P. aeruginosa* containing *sacB* gene was performed by streaking a colony on LB (no salt) + 10% sucrose at 30°C for 24 h.

### Strain constructions

#### *ΔrsmA* construction

Primers *rsmA*-1 and *rsmA*-2 amplify a 400 bp fragment, which was cloned into pEX18 Tc using an EcoRI site on the primer, and a BamHI in the genomic DNA sequence. Primers *rsmA*-3 and *rsmA*-4 amplify a 200 bp sequence, which was cloned into the vector via BamHI and XbaI. The Gm/GFP cassette from pPS858 was inserted into the BamHI site. Upon allelic replacement, the resistance cassette was subsequently excised with FLP recombinase via introduction of pFLP2 plasmid (Hoang *et al*., 1998). The resulting mutation deletes the first 70 bp of *rsmA*, and 55 bp of the upstream region.

#### *Δpel* and *Δpsl* construction

Construction of Δ*pel* and Δ*psl* strains were performed as previously described (Kirisits *et al*., 2005; Starkey *et al*., 2009).

#### pRsmA ox construction

A 281 bp fragment of *rsmA* gene and 66 bp upstream of the start codon generated by PCR using primers *rsmA* EcoRI and *rsmA* XbaI was cloned into pUCP18. Resulting pRsmA ox plasmid and its vector control pUCP18 were electroporated into *P. aeruginosa* as described previously (Choi *et al*., 2006).

#### *lacZ* translation fusion plasmid mini-CTX *lacZ* EB construction

mini-CTX *lacZ* EB was constructed by ligating SmaI/SacI digested mini-CTX *lacZ* backbone with SmaI/SacI digested pMC1403 fragment.

#### Transcriptional and translational fusion construction

Upstream promoter region of *pslA* was amplified by PCR using oligonucleotides listed in Table S2. PCR products, mini-CTX *lacZ* (for transcriptional fusion) and mini-CTX *lacZ* EB (for translational fusion) were digested with EcoRI/BamHI, and ligated. The resulting plasmids were conjugated into *P. aeruginosa* strains and the constructs integrated into the *attB* site as described previously (Hoang *et al*., 2000). mini-CTX plasmid backbone was removed upon expressing FLP recombinase via pFLP2, as β-galactosidase was not efficiently expressed when the backbone DNA was present for unknown reasons (data not shown).

#### RsmA–His6 overexpression vector construction

*rsmA*–His_6_ was amplified by PCR using oligonucleotides *rsmA*–His_6_ for and *rsmA*–His_6_ rev. EcoRI/XbaI digests of *rsmA*–His_6_ and pUCP18 were ligated and introduced into *P. aeruginosa* by electroporation (Choi *et al*., 2006).

### 5′ RACE

The Invitrogen 5′ RACE System was utilized for cDNA synthesis and PCR as per manufacturer's instructions for the high GC content genome using primers *pslA*–GSP1 and *pslA*–GSP2 (Table S2). RNA was isolated from PAO1 cultures grown in LB to OD_600_ ≈ 1.0.

### β-Galactosidase assays

β-Galactosidase activity was quantitatively assayed using a Galacto-Light Plus kit as described elsewhere (Whiteley *et al*., 1999; Lequette *et al*., 2006). *P. aeruginosa* strains were grown in 15 ml of VBMM at 37°C with 250 r.p.m. shaking. One millilitre culture aliquots were extracted, and 200 µl chloroform was added immediately and vortexed for approximately 10 s. Cell lysates were assayed for both β-galactosidase activities, as well as for protein content by Bradford assay (Bio-Rad). All β-galactosidase activity units are normalized by total protein per ml aliquots. All assays were done in triplicates.

### Quantitative real-time PCR

*Pseudomonas aeruginosa* strains were grown in VBMM at 37°C with aeration and monitored for OD_600_ during growth. A 0.5 ml culture was harvested at mid-log phase (OD_600_ ≈ 0.3) and at stationary phase (OD_600_ ≈ 0.9) and treated with RNAprotect reagent (Qiagen) before storing at −80°C. RNA was extracted using RNeasy Mini Kit (Qiagen) according to the manufacturer's protocol (enzymatic lysis and mechanical disruption protocol) with on-column DNase digestion option. RNA concentration and purity were determined by measuring OD_260_, OD_230_ and OD_280_. One microgram RNA was further subjected to DNase I treatment (Promega) at 37°C for 2 h. Complete digestion of genomic DNA was confirmed by PCR using *rplU* forward and *rplU* reverse primers as described previously (Schuster *et al*., 2004). cDNA synthesis was performed using SuperScript III First-Strand Synthesis (Invitrogen) according to the manufacturer's protocol using random hexamers. Eighty-four microlitres of H_2_O was added to the cDNA samples to prevent interference of real-time PCR by reagents from the reverse transcription reactions. Quantitative real-time PCR was performed with 7500 Real-Time PCR System with the SYBR Green PCR Master Mix (Applied Biosystems) as previously described (Borlee *et al*., 2010). Standard curves for transcript quantification was generated using genomic DNA of PAO1 ranging from 2 ng µl^−1^ to 0.02 pg µl^−1^. Primers are listed in Table S2 (*ampR*-F and *ampR*-R, *pslA*-F1 and *pslA*-R1). *pslA* transcript levels were normalized to *ampR* transcript levels. Quantitative real-time PCR was repeated for cDNA synthesized from three or more biologically independent cultures.

### α-Psl immunoblots

Psl immunoblots were performed as previously described (Byrd *et al*., 2009) with the following changes. One millilitre of *P. aeruginosa* VBMM cultures was harvested at respective growth phases. Cells were centrifuged, and the pellet was resuspended in 100 µl of 0.5 M EDTA. Alternatively, cells were collected from tryptone Congo Red agar surfaces grown as previously described (Güvener and Harwood, 2007). Cells were boiled at 100°C for 30 min and centrifuged. The supernatant fraction was subjected to the crude polysaccharide extraction as previously described, while the pellet was resuspended in 1 ml of 6 M urea, boiled for 60 min and assayed for protein concentration by Bradford assay (Bio-Rad). Polysaccharide preparations were diluted and normalized with 0.5 M EDTA to equal protein contents as assayed by Bradford. Five microlitres of polysaccharide preparations were spotted onto a nitrocellulose membrane. Blocking step was performed using 5% milk solution in TBST for 1 h at room temperature, and then probed using α-Psl antibodies in TBST + 1% milk for 1 h at room temperature. Secondary antibodies used in this study were horseradish peroxidase (HRP)-conjugated goat anti-rabbit IgG used at 1:10 000 in TBST (Pierce) for 1 h at room temperature. Densitometry analysis was done using ImageQuant software (Molecular Dynamics).

### Colony morphology photographs

For determination of colony morphologies, strains were streaked on VBMM 1% Noble agar plates supplemented with 40 µg ml^−1^ Congo Red and 15 µg ml^−1^ Coomassie Brilliant Blue R at 37°C for 2 days. Colonies were photographed using a digital camera mounted on a dissection microscope (Olympus SZX-ILLK100).

### α-RsmA Western blots

α-RsmA Western blots were performed using antibodies raised against *E. coli* CsrA (Gudapaty *et al*., 2001). Briefly, *P. aeruginosa* strains were grown in LB shaking cultures at 37°C to their respective growth phases and lysed by addition of perchloric acid to 0.6 M final concentration. Lysed cells were stored on ice for 30 min and centrifuged at 15 000 *g* for 10 min at 4°C. Resulting pellet was resuspended in 6 M urea, boiled and assayed for protein concentration by Bradford assay (Bio-Rad) for normalization before being loaded onto an 18% Tris-HCl polyacrylamide gel. Following electroblotting onto a PVDF membrane, RsmA proteins were detected using α-CsrA antibodies at 1:5000 dilution and 1:20 000 diluted goat α-rabbit HRP-conjugated secondary antibodies (Pierce).

### RsmA overexpression and purification

As BL21(DE3) cells expressed RsmA–His_6_ at a poor level, PAO1 expressing RsmA–His_6_ was grown to stationary phase in 1 litre 2× YT (16 g tryptone, 10 g yeast extract, 5 g NaCl per litre) supplemented with 300 µg ml^−1^ carbenicillin at 37°C with shaking in a baffled 2 l flask. Cells were pelleted at 4°C and stored at −20°C. Cell pellets were resuspended in 10 ml of buffer (50 mM Tris-HCl, 0.5 M NaCl, 10 mM imidazole, 10% glycerol, 5 mM β-mercaptoethanol, pH 7.5) and treated with 1 mg ml^−1^ lysozyme for 30 min on ice. Cells were not treated with DNase I or RNase A as the addition of these enzymes severely reduced the yield of RsmA–His_6_. Further lysis was performed using microtip sonicator, and cell lysates were centrifuged at 13 000 *g* at 4°C for 1 h. The soluble fractions were loaded onto a HisTrap HP 1 ml column (GE Healthcare) and eluted using the Äkta FPLC system (GE Healthcare). RsmA–His_6_ typically eluted in two fractions: between 160–240 and 300–500 mM imidazole. The elution fractions from higher imidazole concentration was found to be pure RsmA–His_6_ and confirmed by Western blotting to the His_6_ tag using HisProbe kit (Pierce) and CsrA antisera (Liu *et al*., 1997) (Fig. S4). The elution fractions were pooled and dialysed against 1 litre 10 mM MgCl_2_, 10 mM Tris-HCl, 100 mM KCl, 25% glycerol, pH 8.0 for 2 h, 3 h and overnight at 4°C. A large portion of RsmA–His_6_ was found to be precipitated post dialysis. RsmA–His_6_ was quantitated by Bradford assay (Bio-Rad) before being aliquoted to 20 µl volumes and stored at −80°C.

### RNA gel mobility shift assay

Quantitative gel mobility shift assays followed a previously published procedure (Yakhnin *et al*., 2007). DNA templates for generating *psl*and *psl* GG→CC RNA transcripts were PCR amplified using primers *pslA*-T7-F and *pslA*-T7-R resulting in a 171 bp product. RNA was synthesized *in vitro* using the MEGAshortscript kit (Ambion) using the PCR products for *psl* and *psl*GG→CC. *phoB* RNA was synthesized as previously described (Jonas *et al*., 2009). After gel purification, transcripts were 5′-end labelled using T4 polynucleotide kinase and [γ-^32^P]-ATP. Radiolabelled RNA was gel purified and resuspended in TE (10 mM Tris-HCl pH 8.0, 1 mM EDTA), heated to 85°C and chilled on ice. Increasing concentrations of purified RsmA–His_6_ recombinant protein were combined with 80 pM radiolabelled RNA in 10 µl binding reactions [10 mM Tris-HCl pH 7.5, 10 mM MgCl_2_, 100 mM KCl, 3.25 ng total yeast RNA, 20 mM DTT, 7.5% glycerol, 4 U SUPERasin (Ambion)] for 30 min at 37°C to allow for RsmA–RNA complex formation. Competition assays were performed in the absence or presence of unlabelled RNA specific and non-specific competitor. Binding reactions were separated using 10% native polyacrylamide gels, and radioactive bands were visualized with a phosphorimager (Molecular Dynamics). Free and bound RNA species were quantified with ImageQuant Software (Molecular Dynamics), and an apparent equilibrium binding constant (K*_d_*) was calculated for RsmA–RNA complex formation according to a previously described cooperative binding equation (Mercante *et al*., 2006).
