# Supplementary material for: Pseudomonas aeruginosa biofilm matrix polysaccharide Psl is regulated transcriptionally by RpoS and post-transcriptionally by RsmA
Source: Mol Microbiol. 2010 Sep 2;78(1):158–72. doi: 10.1111/j.1365-2958.2010.07320.x (PMC2984543; doi:10.1111/j.1365-2958.2010.07320.x)
Supplement: Supplementary file 1 [file mmi0078-0158-SD1.pdf]

SUPPLEMENTARY MATERIAL

PAGES CONTENTS

1-2 Supplementary Text
3-6 Supplementary Tables 1 to 2
7-13 Supplementary Figures 1 to 9
14 Supplementary References

SUPPLEMENTARY TEXT

Δ*rsmA* mutants have previously been reported to be slightly slower in growth during log phase than wild type strains (Pessi *et al.*, 2001), but no reports have been published regarding other growth phases. We observed consistently slower growth in liquid culture, and smaller colony sizes of Δ*rsmA* compared to Δ*wspF*, but the slight changes in growth during log phase do not fully account for this difference. We therefore monitored the growth of these strains throughout their culturing. Due to the highly auto-aggregative nature of Δ*rsmA*, accurately measuring the optical density of the liquid cultures was not possible. We therefore used a Δ*rsmA* Δ*pel* Δ*psl* strain, which does not auto-aggregate (data not shown). Consistent with the previous report, only a slight difference was observed in the doubling time of Δ*rsmA* Δ*pel* Δ*psl* mutant compared to PAO1 wild-type and Δ*pel* Δ*psl* mutant (**Fig. S7**). However, the Δ*rsmA* strain was defective in growth during lag phase, delaying its arrival at log and stationary phases by 2-3 h compared to wild-type strains. Mulcahy *et al.* (2008) reported that PAO1 wild-type out-competed Δ*rsmA* by 4 hours of co-culture. This could be due to the extended lag phase of Δ*rsmA*.

Pessi *et al.* (2001) reported that Δ*rsmA* mutants exhibited enhanced production of the extracellular pigment pyocyanin. We, however, observed a complete lack of pigment production (pyocyanin and pyoverdine) in Δ*rsmA* compared to PAO1 wild-type (**Fig. S8**). Furthermore, we observed an identical pigment defect phenotype in Δ*wspF* and inducible Psl over-expression strains, both of which are auto-aggregative in culture (data not shown). Interestingly, the non-auto-aggregating strains Δ*rsmA* Δ*psl* and Δ*rsmA* Δ*pel* Δ*psl* demonstrated wild-type levels of pigment production (**Fig. S8**), indicating that the pigment phenotype is likely linked to auto-aggregation and is not directly regulated by RsmA.

Secondary site mutation frequencies that give rise to suppressor mutants of Δ*csrA* strains have been documented to be very high in other species (Altier *et al.*, 2000; Timmermans and Van Melderen, 2009). We observed the appearance of suppressor mutants in the Δ*rsmA* background to be high especially in rich medium (50% - 100% suppressor mutants in overnight cultures; **Fig. S9**). Defective growth may be one of the pleoitropic effects that favours the appearance of the suppressor mutants. These suppressors lost the rugose small colony variant (RSCV) morphologies (**Fig. S9**). Interestingly, the suppressors had altered phenotypes in auto-aggregation in liquid cultures and flagella-dependent motility (data not shown). The suppressor mutants do not revert to the RSCV morphologies and are stable, as multiple passages failed to produce RSCVs

(data not shown). These results warn that any future characterization studies done with Δ*rsmA* warrant extreme caution.

SUPPLEMENTARY MATERIAL

SUPPLEMENTARY TABLES

Table S1. Bacterial strains and plasmids.

Strains/plasmids	Description	Source/reference
Pseudomonas aeruginosa		
PAO1	wild type	(Holloway *et al.*, 1979)
Δ*pel*	Δ*pelA*; polar knock-out of *pel* operon	(Borlee *et al.*, 2010)
Δ*psl*	Δ*pslBCD*; polar knock-out of *psl* operon	(Kirisits *et al.*, 2005)
Δ*pel* Δ*psl*		(Borlee *et al.*, 2010)
Δ*rpoS* (MW20)	*rpoS'*::*aacC1*; insertion mutant of *rpoS* Gent^R	(Whiteley *et al.*, 2000)
Δ*wspF*	in-frame deletion mutant of *wspF*	(Hickman *et al.*, 2005)
Δ*wspF* Δ*pel*		this study
Δ*wspF* Δ*psl*		this study
Δ*wspF* Δ*pel* Δ*psl*		(Borlee *et al.*, 2010)
Δ*rsmA*	Δ*rsmA*$_{-57\rightarrow73}$::*FRT*	this study
Δ*rsmA* Δ*pel*		this study
Δ*rsmA* Δ*psl*		this study
Δ*rsmA* Δ*pel* Δ*psl*		this study
Δ*rsmA* rev	suppressor mutant of Δ*rsmA* isolated from overnight LB liquid culture plated on VBMM plate	this study
PAO1 P$_{pslA}$ TR1→DN1	*lacZ* transcriptional fusion construct; *psl*$_{-297\rightarrow+54}$::*lacZ*	this study
Δ*rpoS* P$_{pslA}$ TR1→DN1	*lacZ* transcriptional fusion construct; *psl*$_{-297\rightarrow+54}$::*lacZ* Gent^R	this study
PAO1 P$_{pslA}$ TR1→DN3	*lacZ* transcriptional fusion construct; *psl*$_{-297\rightarrow-87}$::*lacZ*	this study
PAO1 P$_{pslA}$ TR2→DN1	*lacZ* transcriptional fusion construct; *psl*$_{-144\rightarrow+54}$::*lacZ*	this study
PAO1 P$_{pslA}$ TR5→DN1	*lacZ* transcriptional fusion construct; *psl*$_{-393\rightarrow+54}$::*lacZ*	this study
PAO1 P$_{pslA}$ TR0→::*lacZ* trx	*lacZ* transcriptional fusion construct; *psl*$_{-393\rightarrow+8}$::*lacZ*	this study
Δ*pel* Δ*psl* P$_{pslA}$ TR0→::*lacZ* trx	*lacZ* transcriptional fusion construct; *psl*$_{-393\rightarrow+8}$::*lacZ*	this study
Δ*rsmA* P$_{pslA}$ TR0→::*lacZ* trx	*lacZ* transcriptional fusion construct; *psl*$_{-393\rightarrow+8}$::*lacZ*	this study
Δ*rsmA* Δ*pel* Δ*psl* P$_{pslA}$ TR0→::*lacZ* trx	*lacZ* transcriptional fusion construct; *psl*$_{-393\rightarrow+8}$::*lacZ*	this study
PAO1 P$_{pslA}$ TR0→::*lacZ* trl	*lacZ* translational fusion construct;	this study

	$psl_{-393\rightarrow+8}$::'*lacZ*	
Δ*pel* Δ*psl* P_{pslA} TR0→::*lacZ* trl	*lacZ* translational fusion construct; $psl_{-393\rightarrow+8}$::'*lacZ*	this study
Δ*rsmA* P_{pslA} TR0→::*lacZ* trl	*lacZ* translational fusion construct; $psl_{-393\rightarrow+8}$::'*lacZ*	this study
Δ*rsmA* Δ*pel* Δ*psl* P_{pslA} TR0→::*lacZ* trl	*lacZ* translational fusion construct; $psl_{-393\rightarrow+8}$::'*lacZ*	this study
Δ*rsmA* rev P_{pslA} TR0→::*lacZ* trl	*lacZ* translational fusion construct; $psl_{-393\rightarrow+8}$::'*lacZ*	this study
PAO1 P_{pslA} TR0→ GG→CC::*lacZ* trx	*lacZ* transcriptional fusion construct; $psl_{-393\rightarrow+8\ (-30—29\ \mathrm{GG\rightarrow CC})}$::*lacZ*	this study
Δ*rsmA* P_{pslA} TR0→ GG→CC::*lacZ* trx	*lacZ* transcriptional fusion construct; $psl_{-393\rightarrow+8\ (-30—29\ \mathrm{GG\rightarrow CC})}$::*lacZ*	this study
PAO1 P_{pslA} TR0→ GG→CC::*lacZ* trl	*lacZ* translational fusion construct; $psl_{-393\rightarrow+8\ (-30—29\ \mathrm{GG\rightarrow CC})}$::'*lacZ*	this study
Δ*rsmA* P_{pslA} TR0→ GG→CC::*lacZ* trl	*lacZ* translational fusion construct; $psl_{-393\rightarrow+8\ (-30—29\ \mathrm{GG\rightarrow CC})}$::'*lacZ*	this study
PAO1 P_{pslA} no ATG::*lacZ* trx	*lacZ* transcriptional fusion construct; $psl_{-393\rightarrow-1}$::*lacZ*	this study
PAO1 P_{pslA} no ATG::*lacZ* trl	*lacZ* transcriptional fusion construct; $psl_{-393\rightarrow-1}$::'*lacZ*	this study
PAO1 P_{pslA} anti-SD mutant::*lacZ* trx	see Fig. 11	this study
Δ*rsmA* P_{pslA} anti-SD mutant::*lacZ* trx	see Fig. 11	this study
PAO1 P_{pslA} anti-SD mutant::*lacZ* trl	see Fig. 11	this study
Δ*rsmA* P_{pslA} anti-SD mutant::*lacZ* trl	see Fig. 11	this study
Escherichia coli		
DH5α	cloning strain F'/*endA1 hsdR17*($r_k^-m_k^+$) *glnV44 thi-1 recA1 gyrA* (Nal^R) *relA1* Δ*(lacIZYA-argF)U169 deoR* (φ80*dlac*Δ*(lacZ)M15*)	Gibco/BRL
NEB5α	cloning strain *fhuA2* Δ*(argF-lacZ)U169 phoA glnV44* φ*80*Δ(*lacZ*)M15 *gyrA96 recA1 relA1 endA1 thi-1 hsdR17*	New England BioLabs
S17-1 λ*pir*	conjugation donor *recA pro hsdR* RP4-2-Tc::Mu-Km::Tn7 λ*pir*	(Simon *et al.*, 1983)
SM10	conjugation donor *thi thr leu tonA lacY supE recA*::RP4-2-Tc::Mu Km^R	(Miller and Mekalanos, 1988)
**Plasmids**		
pMPSL-KO1	*pslBCD* deletion/polar mutation construct Amp^R $Gent^R$	(Kirisits *et al.*, 2005)
pMPELA	*pelA* deletion/polar mutation construct Amp^R $Gent^R$	(Starkey *et al.*, 2009)
pEX1.8	Broad host range vector Amp/$Carb^R$	(Pearson *et al.*, 1997)
pMW105	pEX1.8::P_{tac}::*rpoS*	(Whiteley *et al.*,

	Amp/Carb^R	2000)
pEX18 Tc	*P. aeruginosa* suicide vector Tet^R	(Hoang *et al.*, 1998)
pPS858	FRT-cassette vector, *gfp*-*aacC1* Gent^R	(Hoang *et al.*, 1998)
pΔ*rsmA* Gm	*rsmA* deletion construct (pEX18 Tc backbone) Tet^R Gent^R	this study
pFLP2	FLP recombinase expressing plasmid Amp/Carb^R	(Hoang *et al.*, 1998)
pUCP18	*P. aeruginosa* - *E. coli* shuttle vector Amp/Carb^R	(Schweizer, 1991)
pRsmA ox	RsmA over-expression plasmid (pUCP18 backbone) Amp/Carb^R	this study
pUCP18::*rsmA*-His$_6$	RsmA-His$_6$ over-expression plasmid Amp/Carb^R	this study
pWspF (pSP5)	WspF over-expression plasmid (pUCP18 backbone) Amp/Carb^R	(Starkey *et al.*, 2009)
pMC1403	*lac'ZYA* Amp^R	(Casadaban *et al.*, 1983)
mini-CTX *lacZ*	*lacZ* transcriptional fusion *attB* integration construction plasmid Tet^R	(Becher and Schweizer, 2000)
mini-CTX *lacZ* EB	*lacZ* translational fusion *attB* integration construction plasmid Tet^R	this study

Table S2. Oligonucleotide primers. Engineered restriction sites are underlined.

Oligonucleotide primer	Sequence (5' to 3')
TR1	GGGAATTCCCCAGGCCAGAGCGCTCGCGG
TR2	GGGAATTCAGCCACTATCGACGAATGAACC
TR5	GGGAATTCCTAAATTGACGCTTCACCGCC
DN1	GATCGGATCCCTCGATGGAATCCAGCCCGTG
DN3	CGGGATCCATTTGCCACGCGGTTTAGT
P2-UTR-RBS-*pslA*' rev2	GATCGGATCCGAATGCATGTTGTTTGCTCTGCCG
P*pslA* GGA-CCA round 1 rev1	GGATTGAGTGGATAGGGAAGAGC
P*pslA* GGA-CCA rev1	GATCGGATCCGAATGCATGTTGTTTGCTCTGCCGATCA<br>CGGGCAGTGGATTGAGTGGATAGGG AAG
P*pslA* no ATG rev	GATCGGATCCGAGTTGGTTTGCTCTGCCGATCACG
3' *pslA* UTR PstI	GCATGTTGTTTGCTCTGCCGATCACGGGCAGTCCATT<br>GAGTGGATAGGGACTGCAGAGGCGGTGAAGCGTC
TGCTCT to PstI rev1	CTGCAGAGGCGGTGAAGCGTC
rsmA-1	CCGAATTCGCAGACCGCCGCCAACATCG
rsmA-2	GCGGATCCGCAGAAATCAAGAGCG
rsmA-3	GGCTAGACACGGGGCGCATGATACC
rsmA-4	GCGGATCCTCAAAGGGAACCAGGTGCGC
rsmA EcoRI	AAGAATTCCGCTCTTGATTTCTGC
rsmA XbaI	GCTCTAGATTAATGGTTTGGCTCTTG
rsmA-His$_6$ for	CGGCGAATTCTGCGGATCCGCCGCCATTTC
rsmA-His$_6$ rev	GCTCTAGATCAGTGGTGGTGGTGGTGGTGATGGTTTG<br>GCTCTTGATC
pslA-GSP1	AGGTACATGCCGCGTTTCAT
pslA-GSP2	ACGTCCAGTGCCTGGAACATAA
rplU forward	CGCAGTGATTGTTACCGGTG
rplU reverse	AGGCCTGAATGCCGGTGATC
ampR-F	GCGCCATCCCTTCATCG
ampR-R	GATGTCGACGCGGTTGTTG
pslA-F1	AAGATCAAGAAACGCGTGGAAT
pslA-R1	TGTAGAGGTCGAACCACACCG
pslA-T7-F	TAATACGACTCACTATAGGGACAAAGCCACTATCGACG<br>AAT
pslA-T7-R	CATGTTGTTTGCTCTGCCGA

SUPPLEMENTARY FIGURES

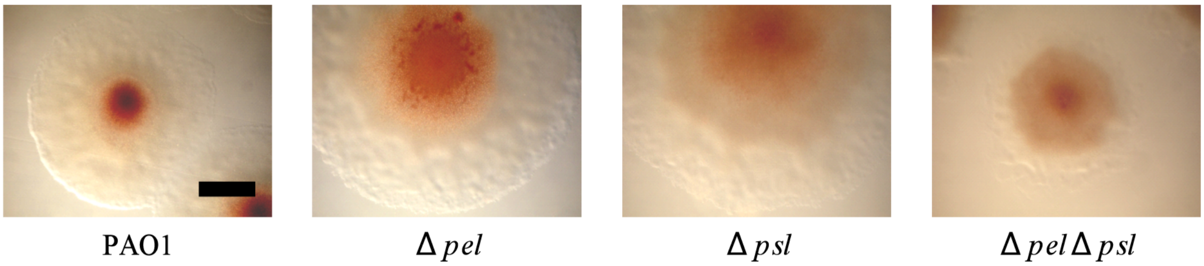

Fig. S1. PAO1 wild-type, Δ*pel*, Δ*psl*, and Δ*pel* Δ*psl* strains all confer smooth colony morphologies. All strains were streaked on VBMM Congo Red plates. Scale bar = 1mm.

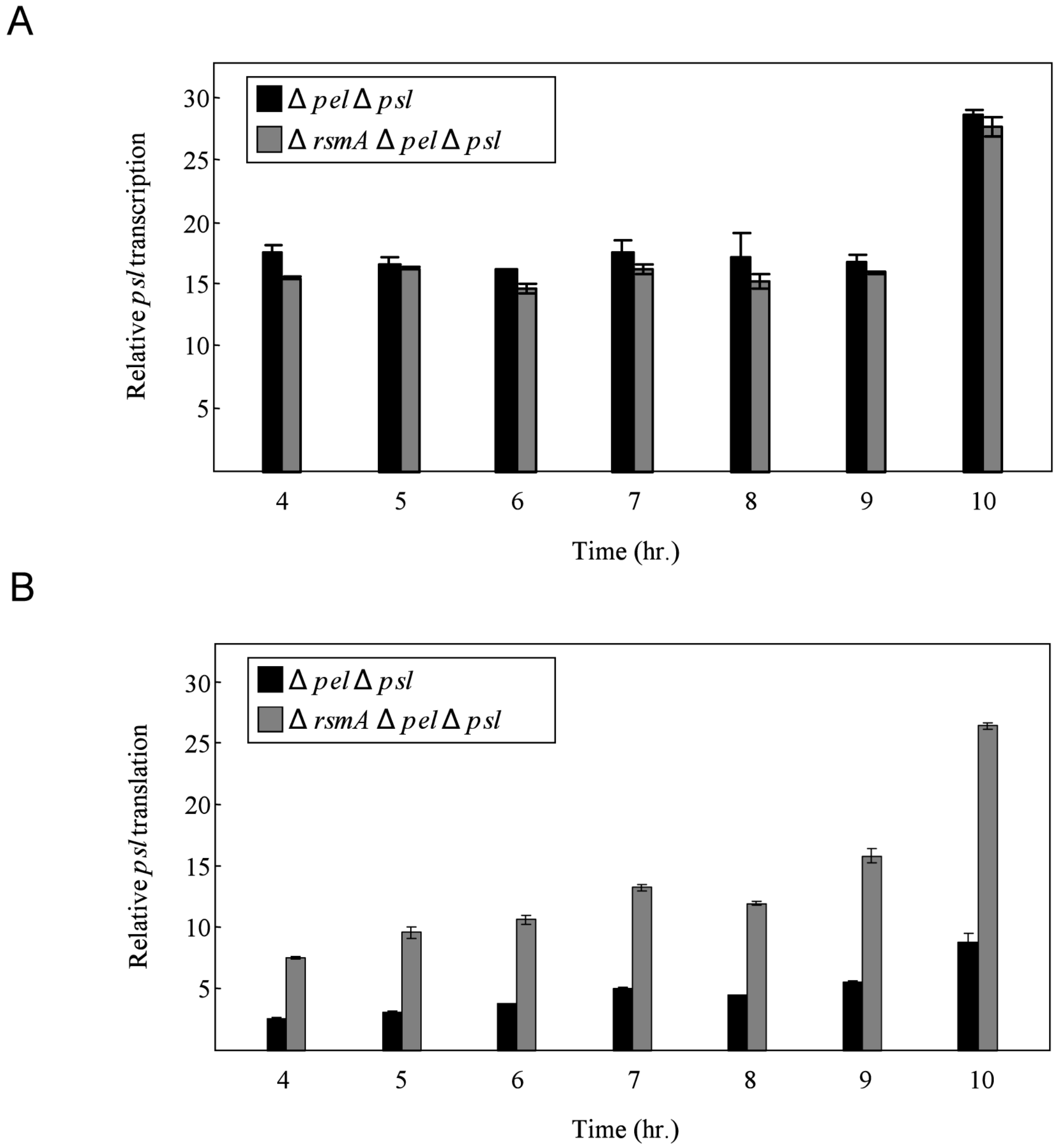

Fig. S2. The effect of *rsmA* mutation on *psl* transcription and *psl* translation is consistent across the growth curve.

A. Transcriptional activities of *psl* in Δ*pel* Δ*psl* and Δ*rsmA* Δ*pel* Δ*psl* are identical across the growth curve. The y-axis unit is described as β-galactosidase activity divided by total mg of protein from the cell lysates.

B. Translational activities of *psl* in Δ*rsmA* Δ*pel* Δ*psl* are consistently higher by ~2-3 fold than Δ*pel* Δ*psl* across the growth curve. The y-axis unit is described as β-galactosidase activity divided by total mg of protein from the cell lysates.

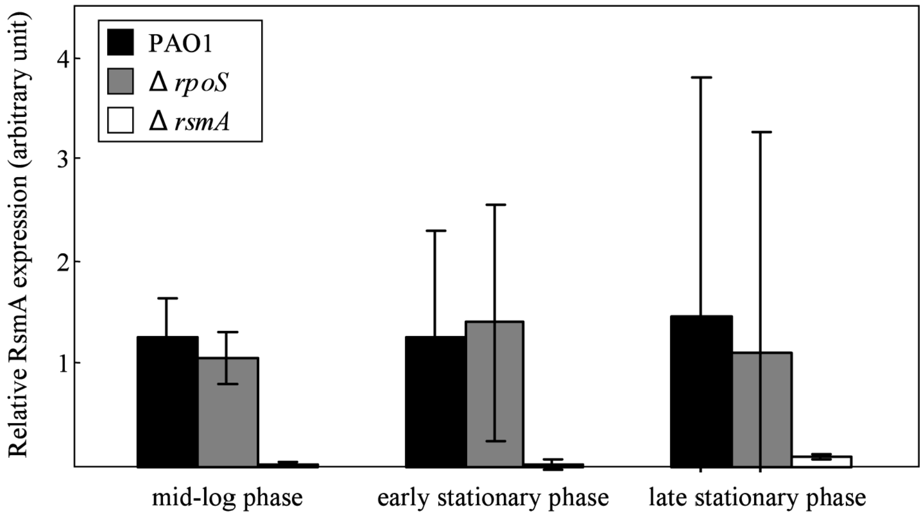

Fig. S3. RsmA protein expression levels are not significantly different throughout culture growth. Densitometry analyses were performed on Western blots shown in Fig. 7, representative of five biological replicates. The y-axis unit is calculated as the ratio of average RsmA signal to loading control signal.

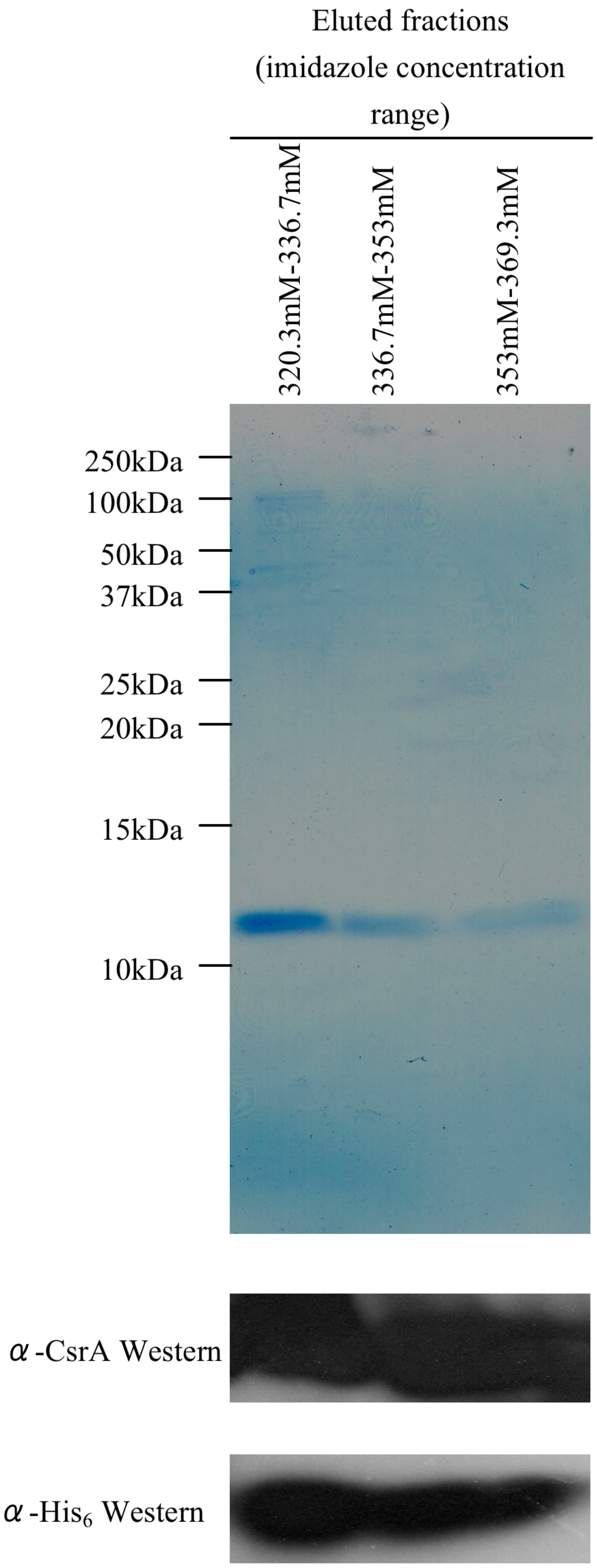

Fig. S4. RsmA-His_6 eluted from the Ni-NTA column at high millimolar range of imidazole with high purity. Western blot analyses using antibodies raised against *E. coli* CsrA and nickel-activated derivative as His_6 motif-specific probe (HisProbe-HRP, Pierce) confirmed that the protein band on Coomassie-stained SDS polyacrylamide gel (top panel) is RsmA-His_6.

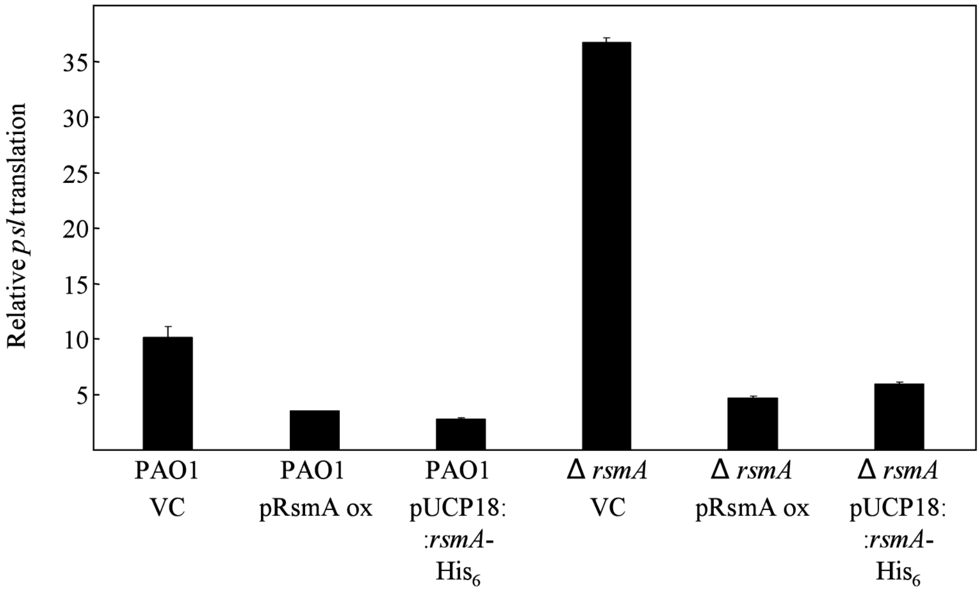

Fig. S5. RsmA-His$_6$ construct is fully active *in vivo* compared to wild-type RsmA. Over-expression of RsmA-His$_6$ showed identical translational *psl* activity compared to wild-type RsmA over-expression. RsmA-His$_6$ was able to complement Δ*rsmA*, and reduce *psl* translation lower than PAO1 level. The y-axis unit is described as β-galactosidase activity divided by total mg of protein from the cell lysates. VC = vector control (pUCP18).

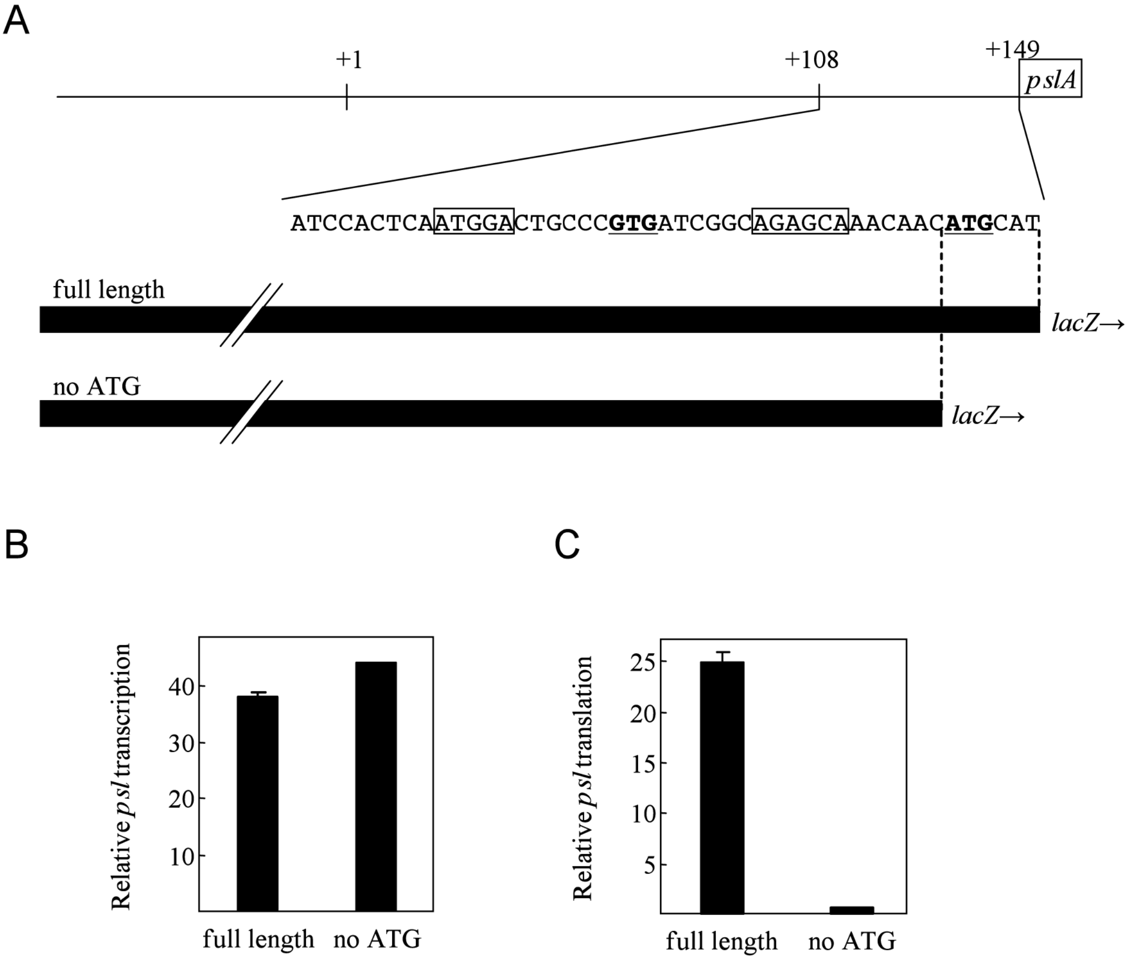

Fig. S6. *pslA* translation most likely starts from the AUG start codon as annotated, not from the GUG start codon located 21 bases upstream.

A. Translational fusion constructs of full length and no ATG constructs. The full length construct contains both putative translational start sites (underlined). The no ATG construct is translationally fused to *'lacZ* just in front of the AUG start codon. This construct contains the alternative translational start site (GTG, underlined) and its putative SD sequence (boxed).

B. The constructs shown in (A) were ligated into transcriptional *lacZ* fusion vector mini-CTX *lacZ* and assayed for β-galactosidase activity. Both constructs had comparable transcriptional activities. The y-axis unit is described as β-galactosidase activity divided by total mg of protein from the cell lysates.

C. Translational activity of the no ATG construct was below the detection limit, suggesting that the AUG codon is essential for translational activity of the construct.

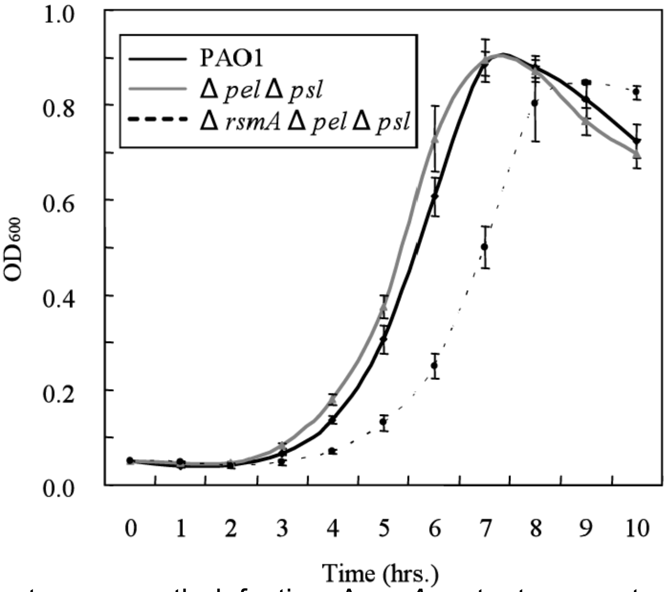

Fig. S7. Δ*rsmA* mutants are growth defective. Δ*rsmA* mutants are auto-aggregative in liquid culture, rendering optical density measurements inaccurate. We therefore compared the growth curve between wild-type and Δ*rsmA* in the Δ*pel* Δ*psl* strain background, which do not aggregate in liquid. Δ*rsmA* mutants appear to have a similar doubling time in log phase and final cell density at stationary phase, but are defective in recovering from lag phase. Data sets were compiled from three independent cultures for each strain grown in VBMM at 37°C.

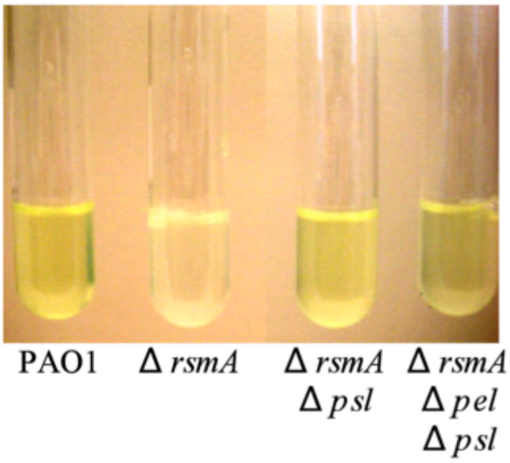

Fig. S8. Loss of pigment production in Δ*rsmA* is dependent on cellular auto-aggregation. Strains were grown in VBMM overnight at 37°C. Photographs of the cultures indicate that non-aggregating strains Δ*rsmA* Δ*psl* and Δ*rsmA* Δ*pel* Δ*psl* are fully proficient in pigment production at wild-type level, indicating that pigment biosynthesis is not under RsmA regulation, but is repressed when cells are auto-aggregating.

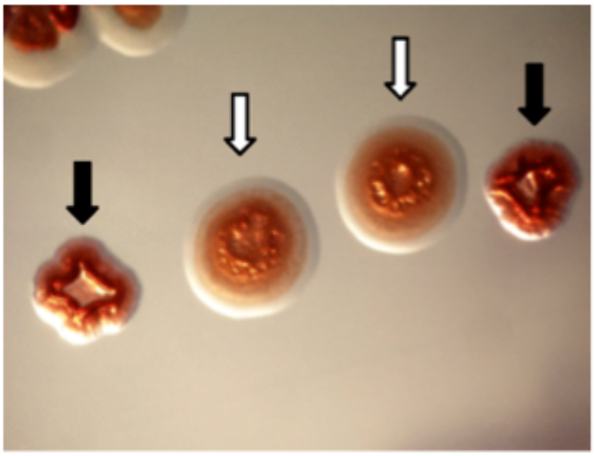

Fig. S9. Δ*rsmA* suppressor mutants arise at high frequency during culture. Δ*rsmA* was grown overnight in LB broth and plated on VBMM + Congo Red. Black arrows indicate parental Δ*rsmA* RSCV colonies while white arrows indicate suppressor mutants (Δ*rsmA* rev) that have lost the RSCV properties on the plate. The Δ*rsmA* rev phenotype is stable, as multiple passages do not give rise to a RSCV phenotype (data not shown).

REFERENCES

Altier, C., Suyemoto, M., and Lawhon, S.D. (2000) Regulation of *Salmonella enterica* serovar typhimurium invasion genes by *csrA*. *Infect Immun* **68**: 6790-6797.

Mulcahy, H., O'Callaghan, J., O'Grady, E.P., Maciá, M.D., Borrell, N., Gómez, C., Casey, P.G., Hill, C., Adams, C., Gahan, C.G., Oliver, A., and O'Gara, F. (2008) *Pseudomonas aeruginosa* RsmA plays an important role during murine infection by influencing colonization, virulence, persistence, and pulmonary inflammation. *Infect Immun* **76**: 632-638.

Pessi, G., Williams, F., Hindle, Z., Heurlier, K., Holden, M.T., Cámara, M., Haas, D., and Williams, P. (2001) The global posttranscriptional regulator RsmA modulates production of virulence determinants and *N*-acylhomoserine lactones in *Pseudomonas aeruginosa*. *J Bacteriol* **183**: 6676-6683.

Timmermans, J., and Van Melderen, L. (2009) Conditional essentiality of the *csrA* gene in *Escherichia coli*. *J Bacteriol* **191**: 1722-1724.
